# Development and validation of the Integrative Vitality Scale

**DOI:** 10.3389/fpubh.2024.1452068

**Published:** 2024-11-18

**Authors:** Seok In Yoon, Hui Yeong Park, Sun Yong Chung, Jong Woo Kim

**Affiliations:** ^1^Department of Neuropsychiatry, College of Korean Medicine, Kyung Hee University, Seoul, Republic of Korea; ^2^Department of Neuropsychiatry, Kyung Hee University Korean Medicine Hospital at Gangdong, Seoul, Republic of Korea

**Keywords:** Integrative Vitality Scale, physical vitality, psychological vitality, traditional eastern medicine, self-determination theory, sustainable well-being

## Abstract

**Introduction:**

Vitality is a construct based on traditional vitalism, and is a concept similar to energy (Qi), passion, and motivation as the essential power possessed by organisms. Recently, various methods and tools have been designed to evaluate vitality as a health indicator. This study aimed to develop and validate an Integrative Vitality Scale (IVS) based on traditional Eastern medicine and modern psychology.

**Methods:**

We conducted two online surveys and one pre-post comparison with Korean adults. Descriptive statistics and factor analysis were performed for scale development, and correlation and regression analysis were performed for validation.

**Results:**

Exploratory (*n* = 348) and confirmatory (*n* = 349) factor analyses showed that two subfactors (physical and psychological vitality) best represented integrative vitality. The IVS-total and subscales had good internal consistency (*α* = 0.89–.094) and test-retest reliability (*r* = 0.71–0.80). Ten health-related experts (e.g., doctors, clinical psychologists, and counselors) evaluated the IVS as having excellent content validity. The IVS-total and subscales had a high correlation with existing vitality-related scales but a low correlation with pathological symptoms such as hypomania, suggesting convergent and discriminant validity. The IVS-total and subscales were negatively correlated with depression and fatigue but positively correlated with well-being and quality of life, suggesting criterion validity. The IVS had additional predictive power for depression, fatigue, and well-being even after controlling for existing vitality-related scales, suggesting incremental validity. Finally, after 16 weeks of mindfulness training (*n* = 28), IVS-total and subscales significantly increased.

**Discussion:**

These findings suggested that the IVS is a valid and reliable tool for assessing physical and psychological vitality. Furthermore, the IVS could be used as a clinical indicator to predict symptoms related to low energy, such as depression and fatigue, and as an indicator of sustainable well-being.

## 1 Introduction

Healthy aging is a key agenda for achieving the UN Nations’ Sustainable Development Goals in the 2020s, which suggest that quality of life and well-being during aging have emerged as important concerns when the world is entering an aging society. Healthy aging is defined as the process of developing and maintaining functional ability to enable well-being in older age (1). As one of the global strategies to achieve healthy aging, developing effective measurements to assess and monitor healthy aging is required (1). Recently, the World Health Organization (WHO) considered vitality as one of five distinct domains of intrinsic capacity and found that vitality contributes significantly to the development and maintenance of functional ability for healthy aging (2, 3). Therefore, this study aims to develop a measurement to assess vitality, one of the key factors of healthy aging.

The concept of vitality has been linked to the doctrine of vitalism that has been advocated since ancient Greece. In vitalism, vitality refers to the essential force that controls the development of organisms and leads to biology, based on the belief that this force is fundamentally different from the chemical mechanisms of inanimate objects (4). Traditionally, vitality has been used in a manner similar to spiritual energy, enthusiasm, passion, and motivation (5). This suggests that vitality is related to an active lifestyle, a quality originally intended to be expressed externally.

As scientific research progressed, beliefs in vitalism were refuted, and a mechanistic approach to understanding vitality prevailed (5). In recent health-related fields, vitality has been used in the sense of physical and physiological functions or performance close to the mechanistic approach, rather than as the essential force of an organism advocated by vitalism. For example, vitality is often used to refer to physiological levels of function or motor performance. Typically, vital signs, such as pulse, respiration, body temperature, and blood pressure are measured as objective indicators that reflect the state of cardiorespiratory function necessary for maintaining an organism’s life (6). Recently, pain and walking speed have been proposed as the fifth and sixth vital signs, respectively (7, 8). Vitality, which is used to assess motor performance, is measured according to objective motor indicators, such as grip strength and walking speed (7, 9). However, the definition of vitality as a physical and physiological function is limited because it does not contain the original meaning of vitality originating from vitalism. We believe that the mechanistic definition of vitality has made vitality measurable but has diminished its original meaning.

In psychology, vitality is defined as a subjective positive state that is accompanied by physical and psychological energy. Ryan and Frederick (10) conceptualized vitality as a subjective feeling influenced by physical factors. According to Barbic et al. (11), emotional vitality is a multidimensional construct that accompanies physical and psychological well-being, including physical energy, positive mood, mastery, and interest in life. According to Shapiro and Donaldson (12), vitality is an inner resource that can foster an abundance of energy available to the self and is a combination of physical, cognitive, and emotional factors. According to Lavrusheva (13), vitality is a broad and multidimensional concept that includes energy, physical and psychological health, and wellbeing. Vitality accompanies both physical and psychological energy. It is a positive and subjective experience that can be regulated and controlled and is a dynamic resource whose level can vary depending on the context and conditions (13). These definitions are an integrated perspective that encompass positive emotions, motivation, and well-being, unlike perspectives that focus only on physical and physiological functions.

To assess subjective vitality, self-report scales developed to date include the Subjective Vitality Scale (SVS) (10), vitality subdomain of the SF-36 (14), and Leader Vitality Scale (12). However, these scales have a fallacy of circular reasoning, in which the same concepts are asked back to evaluate the vitality or energy experienced by individuals, such as “I feel alive and *vital*,” “I feel *energized*,” and “Did you have a lot of *energy*?” These examples suggest the necessity of developing items based on a more specific operational definition of vitality.

This study aimed to establish a more specific operational definition of vitality and develop an Integrative Vitality Scale (IVS) to comprehensively assess physical and psychological vitality. For this purpose, traditional and modern perspectives on vitality were comprehensively considered. Specifically, physical vitality was inspired by the Qi concept of traditional Eastern medicine (TEM), and psychological vitality was inspired by Self-determination theory (SDT).

### 1.1 Physical vitality

In TEM, Qi (i.e., Chi, Ki) corresponds to vitality. Qi is a universal principle that explains all phenomena and is the fundamental energy of an organism. Qi refers to the vital force or energy that symbolizes the source of life, creativity, right action, and harmony and is related to physical, psychological, and spiritual health (10).

Although it is difficult to clearly identify the substance of Qi because it has no specific form, it can be experienced as a perceptible and observable phenomenon (15–17). For example, when angry, we may experience a feeling of energy rising upward, whereas when afraid or surprised, we may experience a downward feeling or dissipation of energy, such as a sinking feeling (18). As another example, when we are depressed, our energy is exhausted and we lose our strength and motivation, whereas when we are happy and joyful, our energy is relaxed and we feel comfortable (15, 18). Thus, each feeling is experienced as a unique flow of energy, and these experiences are phenomena of energy that can be observed through introspection.

In recent psychology, balance and harmony have been considered the gold threads of well-being in various domains, including affective, cognitive, behavioral, and relational domains (19). Similarly, in TEM, the core principle of health management is to achieve balance and harmony in Qi (15, 18). According to the Chinese classic Huangdi Nei Jing, seven states of Qi caused by excessive emotions result in a psychophysiological pathology (20). It could be interpreted that physical and psychological pain are caused by disharmony and imbalance of Qi. Considering that vitality is a subjective positive feeling associated with health (10, 11, 13, 21), it could be considered the optimal state in which Qi is harmonized and balanced.

To explain physical vitality, we focused on balancing the autonomic nervous system through a relaxation response. The fight-or-flight response occurs when faced with a situation that induces adaptive behavior and activates the sympathetic nervous system to increase blood pressure, respiratory rate, metabolic rate, and heart rate (22). Conversely, the relaxation response is a survival mechanism opposite to that of the fight-or-flight response (23); it activates the parasympathetic nervous system and reduces blood pressure, respiratory rate, metabolic rate, and heart rate, which are elevated owing to the fight-or-flight response. In other words, the relaxation response is a physiological mechanism for returning to a healthy balance after a stressor and corresponds to the self-recovery power of an organism.

Relaxation and rest are important for maintaining optimal health under excessive and chronic stress. In modern society, competition and obsession with success lead to excessive tension and chronic stress. Chronic stress overactivates the sympathetic nervous system and depletes its physiological resources (24), eventually leading to disorders, such as cardiovascular disease, depression, and anxiety (25–27). Consequently, relaxation and rest are important for restoring autonomic nervous system balance and replenishing vitality (23, 27, 28).

We operationally defined physical vitality as a positive physical experience of relaxation and rest. The physical vitality assessed in this study is not simply whether an individual is performing well but whether the individual is accumulating energy appropriately for the next performance. This definition of vitality is similar to the notion proposed by WHO (e.g., capacity to retain capacity) (3). In other words, physical vitality emphasizes resilience. This definition is distinct from the mechanistic approaches that focus on physical function and performance.

### 1.2 Psychological vitality

If we have replenished energy through relaxation, we should be active again with replenished energy. While physical vitality is related to the recovery of energy through relaxation, psychological vitality is related to making good use of recovered energy.

The SDT provides ideas regarding psychological vitality. The SDT posits that all humans have evolved to become inherently curious, active, vital, and socially connected (29). This suggests that all human beings have an innate propensity to be interested in, explore, and understand both the internal and external worlds, and, furthermore, to actively organize their lives. This view is similar to that of vitalism, which considers vitality to be related to active lifestyles such as enthusiasm and motivation (5).

Motivation is a psychological process that provides energy and direction for action (30). Motivation can be largely classified into three types: amotivation, extrinsic motivation, and intrinsic motivation. This classification is determined by the level of self-determination accompanying a certain action (31, 32). Amotivation refers to the absence of motivation. Extrinsic motivation is divided into four types (extrinsic regulation, introjection regulation, identification regulation, and integrative regulation) according to the degree of autonomy. In extrinsic regulation, people are motivated by the valence of external stimuli and those conditioned by this motivation have difficulty initiating or maintaining tasks in the absence of external stimuli. As motivation is directed toward integrative regulation, the same extrinsic motivation involves more intrinsic autonomy than reliance on external stimuli. Finally, intrinsic motivation refers to motivation that is fully regulated by individual autonomy regardless of external stimuli.

Psychological vitality is associated with an intrinsically motivated state (11) and is sustainable because intrinsic motivation is based on autonomy (29). Because extrinsically motivated behavior is determined by the incentives of external conditions, the behavior stops when the external conditions disappear (30). For example, hypomania is associated with a high behavioral activation system, which implies sensitivity to external rewards (33). Vancampfort et al. (34) showed that goal setting and activities driven by involuntary motivation are difficult to maintain in patients with affective disorders such as bipolar disorder. Conversely, intrinsically motivated behavior is independent of external conditions because it emerges spontaneously from the innate pursuit of psychological needs such as growth and self-actualization (30). One study showed that participants in NGO organizations with voluntary motivations persisted in social movements longer than those with involuntary motivations (35). Accordingly, psychological vitality is the energy that not only “initiates” certain activities but also “sustains” them.

In this study, psychological vitality was operationally defined as a intrinsically motivated state of being active, feeling interested, and having fun in life. Psychological vitality must be distinguished from an impulsive state, which is excessively obsessed with external rewards. This is because excessive obsession and greed cause problems such as addiction, reduced vitality (15), and hinder the growth of human nature (36). In other words, psychological vitality refers to a self-regulated motivational state that is distinct from pathological high-energy states such as mania.

## 2 Methods

### 2.1 Creation of a preliminary item pool

To create a preliminary item pool for the IVS, a literature review, workshops, and semi-structured interviews were conducted. First, we reviewed vitality-related concepts and assessment tools from previous studies in health-related fields such as medicine and psychology (5–14).

Second, three workshops were conducted to establish an integrative model for vitality and create a preliminary item pool. Four Korean medical doctors and two psychologists (PhDs) attended the first workshop on December 13, 2022. The 2nd workshop held on February 4, 2023, was attended by six Korean medical doctors and three psychologists (PhD). The 3rd workshop held on February 24, 2023, was attended by three Korean medical doctors, 11 undergraduate students of Korean Medicine, and two psychologists (PhDs).

Third, semi-structured interviews were conducted to examine the clinical experiences of the health-related experts regarding vitality. Semi-structured interviews were conducted for approximately one hour with six experts in health-related fields, including doctors, Korean medical doctors, clinical psychologists, rehabilitation psychologists, and counseling psychologists.

Consequently, a pool of 36 preliminary items was created. Specifically, it consisted of 19 preliminary items on physical vitality and 17 preliminary items on psychological vitality.

### 2.2 Study 1: content validity

Study 1 was conducted to verify the content validity of the preliminary IVS items.

Content validity is an indicator of whether an assessment tool appropriately represents the content or topic it intends to measure. Ten experts rated the content validity of 36 preliminary items. The expert group consisted of one doctor, six Korean medical doctors, and three psychologists, who were recruited through convenience and snowball sampling.

The experts rated the content validity of each preliminary item from 1 (not at all appropriate) to 4 (very appropriate). The rating criteria were as follows: Does each item match the operational definition of physical or psychological vitality? Second, is each item highly related to an individual’s health and well-being? Third, is each item clear and free of ambiguity? Fourth, is each item easily understandable by the general public? The content validity of each item was rated by comprehensively considering the four criteria.

The content validity evaluation was conducted twice. In the 1st round, the content validity of 36 preliminary items was rated and new ideas (e.g., additional preliminary items) were suggested by experts. In the 2nd round, the content validity of the preliminary items, which were revised and added after the 1st round, was assessed.

Content validity index (CVI) refers to the standards of Polit et al. (37). I-CVI refers to the proportion of experts who responded with a score of 3 or 4 among all experts for each item. In the 1st round, items with an I-CVI between 0.60 and 0.78 were revised or deleted, and items with an I-CVI of less than 0.60 were deleted. In the 2nd round, items with an I-CVI of less than 0.78 were deleted.

The S-CVI/Ave of the final scale was calculated. The S-CVI/Ave was calculated as the average I-CVI of the scale. According to Shi et al. (38), content validity is considered excellent when S-CVI/Ave is higher than 0.90.

### 2.3 Study 2: development of the IVS (sample 1)

#### 2.3.1 Participants and procedure

Study 2 was conducted to determine the number of factors and final IVS items.

Study 2 was conducted as an online survey of 350 Korean adults using Macromill Embrain. Nunnally (39) and Pett et al. (40) suggested that more than 10 subjects per initial variable are needed in factor analysis to reduce sampling error. Accordingly, 350 subjects were sampled to conduct exploratory factor analysis on 32 preliminary items in Study 2. Stratified sampling was performed based on sex and age. Specifically, the sampling ratios of participants in their 20s, 30s, 40s, 50s, and 60s and older were equal, and the gender ratios within each age group were also equal. The survey period was September 22–26, 2023. Participants received 2,000 KRW as a reward for completing the survey. Sample 1 included 348 individuals (174 women) after excluding two outliers. Their average age was 44.74 (*SD* = 14.36).

#### 2.3.2 Measurement

##### 2.3.2.1 IVS preliminary items-32

In Study 2, 32 preliminary IVS items were used. IVS is a 5-point Likert self-report scale, and the score for each item ranges from 0 to 4. After item analysis and exploratory factor analyses (EFA), the number of factors and final items of the IVS were determined.

##### 2.3.2.2 Subjective Vitality Scale (SVS)

To assess subjective vitality, we used the scale developed by Ryan and Frederick (10), the Korean version of which was reconstructed by Kwon (41). The SVS is a 5-point Likert self-report scale consisting of six items. The score for each item ranges from 1 to 5, and the total score ranges from 6 to 30. Higher scores indicate higher subjective vitality. In Study 2, the Cronbach’s *α* was 0.89.

##### 2.3.2.3 Korean version of the Beck Depression Inventory (K-BDI)

To assess depression, we used the scale developed by Beck (42), the Korean version of which was validated by Rhee et al. (43). The K-BDI is a 4-point self-report scale consisting of 21 questions. The score for each question ranges from 0 to 3, and the total score ranges from 0 to 63. Higher scores indicate more depressive symptoms. In a previous study (43), the Cronbach’s *α* was 0.85, and in Study 2, it was 0.91.

##### 2.3.2.4 Fatigue Scale (FS)

The scale developed by Chalder et al. (44) was used to assess fatigue. The FS is a 4-point Likert self-report scale consisting of 14 items. The score for each item ranges from 1 to 4, and the total score ranges from 14 to 56. A higher score indicates more severe fatigue symptoms. In a previous study (45), the Cronbach’s *α* was 0.84, and in Study 2, it was 0.90.

##### 2.3.2.5 Concise Measure of Subjective Well-Being (COMOSWB)

We used the scale developed by Suh and Koo (46) to assess subjective well-being. The COMOSWB is a 7-point Likert self-report scale consisting of nine items, and the score for each item ranges from 1 to 7. The COMOSWB consists of three subscales: life satisfaction, positive emotion, and negative emotion. The total score is calculated as life satisfaction + positive emotion - negative emotion. Higher scores indicate higher subjective well-being. In a previous study (46), the Cronbach’s *α* of the subscales was 0.70–0.83, and the overall Cronbach’s *α* was 0.79. In Study 2, the Cronbach’s *α* of the subscales was 0.84–0.92, and the overall Cronbach’s *α* was 0.92.

##### 2.3.2.6 Korean version of WHO Quality of Life Scale abbreviated version (WHOQOL)

To assess the quality of life, we used a scale developed by the World Health Organization, the Korean version of which was validated by Min et al. (47). The WHOQOL is a 5-point Likert self-report scale consisting of 26 items, and the score for each item ranges from 1 to 5. The WHOQOL consists of five domains: overall quality of life, physical health, psychological health, social relationships, and environment. The average score of each domain is multiplied by 4 to calculate the domain score, and then the total score is calculated as the sum of the domain scores. The score for each domain ranges from 4 to 20, and the total score ranges from 20 to 100. A higher score indicates a higher quality of life. In a previous study (47), the overall Cronbach’s *α* was 0.90, and in Study 2, it was 0.90.

#### 2.3.3 Analysis strategy

Statistical analysis was performed using SPSS 22.0.

To confirm the appropriateness of the items, item analysis was conducted based on descriptive statistics, correlation coefficients, and internal consistency. If the response distribution for each item did not represent a normal distribution, mean or standard deviation was too high or low, inter-item or item-total correlation was too high or low, or reliability of the scale increased when an item was removed, then the corresponding item was excluded.

EFA was conducted to determine the number of factors and final items of the IVS. EFA was conducted using principal axis factoring and the direct oblimin method. To determine the number of subfactors, eigenvalues, scree plots, and interpretability were considered. Items were excluded if they did not strongly load on any factors (all loadings <0.30) or if they cross-loaded on multiple factors (two or more loadings ≥0.30) with loading differences less than 0.10.

After EFA, the final version of the IVS was used for further validation. The convergent and criterion validity of the IVS were evaluated using Pearson’s correlation coefficient. A hierarchical regression analysis was conducted to investigate the incremental validity of the IVS. Incremental validity was evaluated by verifying whether *ΔR*^2^ in Step 2 for depression, fatigue, and subjective well-being was significant when SVS was added in Step 1 and then IVS was added in Step 2.

### 2.4 Study 3: validation of the IVS (sample 2)

#### 2.4.1 Participants and procedure

Study 3 investigated the validity and test–retest reliability of the final version of the IVS.

Study 3 was conducted as an online survey of 350 Korean adults using Macromill Embrain. Tabachnic and Fidell (48) suggested that a minimum sample size of 300 is required for factor analysis, and Comrey and Lee (49) suggested that a total sample size of 300 or more is good. Accordingly, Study 3 sampled 350 subjects. Stratified sampling was performed based on sex and age. Specifically, the sampling ratios of participants in their 20s, 30s, 40s, 50s, and 60s and older were equal, and the gender ratios within each age group were also equal. The respondents in Study 2 were excluded from Study 3. The survey period was November 23–27, 2023. Participants received 2,000 KRW as a reward for completing the survey. Sample 2 included 349 participants (175 women), excluding one outlier. The average age was 44.42 (*SD* = 14.02).

Two surveys were conducted at two-week intervals to investigate the test–retest reliability of the IVS. The resurvey was administered to the first 100 respondents from Sample 2 on a first-come-first-served basis. The re-survey period was December 11–14, 2023. The number of participants in the resurvey was 99 (56 women), after excluding one outlier. The average age was 50.89 (*SD* = 12.96).

#### 2.4.2 Measurement

##### 2.4.2.1 IVS

The IVS developed in Study 2 was used in this study. The IVS is a 5-point Likert self-report scale consisting of 22 items, and the score for each item ranges from 0 to 4. The IVS consists of two subscales: physical and psychological vitality. The total score ranges from 0 to 44 for physical vitality, 0 to 44 for psychological vitality, and 0 to 88 for overall vitality. A higher score indicates a higher level of vitality. The final version of the IVS is presented in Supplementary material 1.

##### 2.4.2.2 Short Form 36 Health Survey – Vitality subdomain (SF-36-VIT)

To assess subjective vitality, we used the scale developed by Ware (50), the Korean version of which was validated by Koh et al. (51). The SF-36-VIT is a 6-point Likert self-report scale consisting of four items. The score for each item ranges from 1 to 6, and the total score ranges from 4 to 24. A higher score indicates a higher level of health. In a previous study (51), the Cronbach’s *α* in the vitality subdomain was 0.65, and in Study 3, it was 0.77.

##### 2.4.2.3 K-BDI

The same scale used in Study 2 was used here. In a previous study (43), the Cronbach’s *α* was 0.85, and in Study 3, it was 0.91.

##### 2.4.2.4 FS

The same scale used in Study 2 was used here. In a previous study (45), the Cronbach’s *α* was 0.84, and in Study 3, it was 0.91.

##### 2.4.2.5 COMOSWB

The same scale used in Study 2 was used here. In a previous study (46), the Cronbach’s *α* of the subscales was 0.70–0.83, and the overall Cronbach’s *α* was 0.79. In Study 3, the Cronbach’s *α* of the subscales was 0.86–0.93, and the overall Cronbach’s *α* was 0.90.

##### 2.4.2.6 Korean version of the Basic Psychological Need Satisfaction Scale (BPNSS)

The scale developed by Deci and Ryan (52) and Gagné (53), the Korean version of which was validated by Lee and Kim (54), was used. The BPNSS is a 6-point Likert self-report scale consisting of 18 items, with the score for each item ranging from 1 to 6. The BPNSS comprises three subscales: autonomy, competence, and relatedness. The total score ranges from 6 to 36 for autonomy, 6 to 36 for competence, 6 to 36 for relatedness, and 18 to 108 overall. Higher scores indicate higher satisfaction with basic psychological needs. In a previous study (54), the Cronbach’s *α* of the subscales was 0.70–0.79, and the overall Cronbach’s *α* was 0.87. In Study 3, the Cronbach’s *α* of the subscales was 0.81–0.92, and the overall Cronbach’s *α* was 0.91.

##### 2.4.2.7 Korean version of the Hypomania Checklist (K-HCL-32)

To assess hypomania, we used the scale developed by Angst et al. (55) and validated as a Korean version by Oh et al. (56). The K-HCL-32 is a dichotomous scale comprising 32 items. Higher scores indicate more severe hypomanic symptoms. In a previous study (56), Cronbach’s *α* was 0.88, and in Study 3, Cronbach’s *α* was 0.78.

#### 2.4.3 Analysis strategy

Statistical analysis was performed using Amos 23.0 and SPSS 22.0.

A confirmatory factor analysis (CFA) was conducted to confirm the two-factor structure of the IVS observed in the EFA. The maximum likelihood method was used to estimate the model. In CFA, model fit was evaluated using *χ^2^* statistic, comparative fit index (CFI), Tucker–Lewis index (TLI), root mean square error of approximation (RMSEA), and standardized root mean square residual (SRMR).

Internal consistency and test–retest reliability were evaluated to determine the reliability of the IVS. Internal consistency was evaluated using Cronbach’s *α*, and test–retest reliability was evaluated using Pearson’s correlation coefficients for IVS across timepoints.

The convergent, discriminant, and criterion validities of the IVS were evaluated using Pearson’s correlation coefficients. Hierarchical regression analysis was conducted to investigate the incremental validity of the IVS. Incremental validity was evaluated by verifying whether *ΔR*^2^ in Step 2 for depression, fatigue, and subjective well-being was significant when the SF-36-VIT was added in Step 1 and then the IVS was added in Step 2.

**Figure 1:**
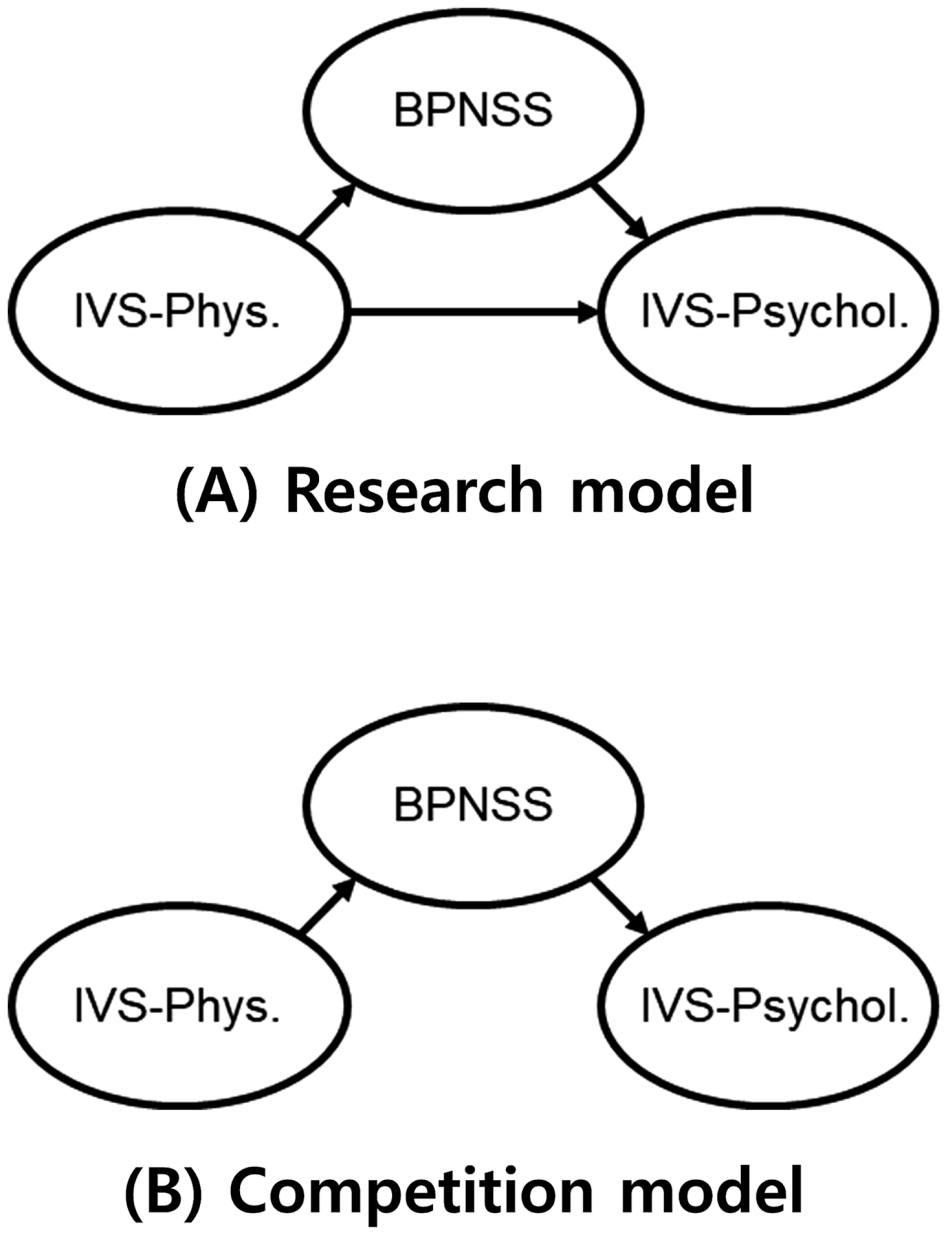
**(A)** the partially mediated model; **(B)** the fully mediated model; IVS-Phys. = Integrative Vitality Scale-Physical vitality; IVS-Psychol. = Integrative Vitality Scale-Psychological vitality; BPNSS = Korean version of the Basic Psychological Need Satisfaction Scale.

A structural equation model was used to determine the construct validity of the IVS. According to the SDT (31, 32), intrinsic motivation is experienced when basic psychological needs, such as autonomy, competence, and relatedness, are satisfied. According to the hierarchy of needs theory (57), in order to pursue basic psychological needs, lower physical and physiological needs such as relaxation and rest must be satisfied first. Accordingly, this study investigated the goodness of fit of a model in which basic psychological needs satisfaction mediated the relationship between physical vitality (i.e., relaxation) and psychological vitality (i.e., intrinsic motivation). Positive feelings promote approach behaviors (58) and induce individuals to actively engage in new environments (59). In other words, physical vitality, which is a positive feeling, may increase psychological vitality in itself. Accordingly, the partially mediated model was set as the research model (Figure 1A) and compared with the fully mediated model (Figure 1B). To compare model fit, the *χ^2^*, CFI, TLI, RMSEA, and SRMR of the research model and the competition model were calculated. Next, we investigated the significance of the coefficients of the direct and indirect paths of the appropriate model.

### 2.5 Study 4: pre-post comparison (sample 3)

#### 2.5.1 Participants and procedure

Study 4 investigated whether IVS could detect changes before and after mindfulness training.

Study 4 was conducted with 31 university students in Republic of Korea who took a course on mindfulness theory and practice. The university students trained breathing meditation, body scan, sitting meditation, and mindful movement (e.g., walking meditation, yoga, qigong) for 16 weeks from March to June 2024. Assessments were conducted at pre and post-training. The analysis was conducted on 28 university students (16 female) who completed all two assessments. The mean age was 21.79 (SD = 2.10).

#### 2.5.2 Measurement

##### 2.5.2.1 IVS

The same scale used in Study 3 was used here.

##### 2.5.2.2 COMOSWB

The same scale used in Study 2 was used here.

##### 2.5.2.3 Psychological Well-Being Scale (PWBS)

To assess psychological well-being, we used the scale developed by Nishida (60), the Korean version of which was validated by Kim and Chang (61). The PWBS is a 7-point Likert self-report scale consisting of 38 items. The score for each item ranges from 1 to 7, and the total score ranges from 38 to 266. The PWBS consists of six subscales: self-acceptance, awareness of goal, autonomy, activeness of life, self-transcendence, and adventure. A higher score indicates a higher level of psychological well-being. In a previous study (61), the Cronbach’s *α* of the subscales was 0.80–0.92, and the overall Cronbach’s *α* was 0.92.

##### 2.5.2.4 Cognitive and Affective Mindfulness Scale-Revised (CAMS-R)

To assess mindfulness, we used the scale developed by Feldman et al. (62), the Korean version of which was validated by Cho (63). The CAMS-R is a 4-point Likert self-report scale consisting of 10 items. The score for each item ranges from 1 to 4, and the total score ranges from 10 to 40. A higher score indicates a higher level of mindfulness. In a previous study (63), the Cronbach’s *α* was 0.70.

#### 2.5.3 Analysis strategy

Statistical analysis was performed using SPSS 22.0. A paired *t*-test was conducted to verify the changes after mindfulness training.

## 3 Results

### 3.1 Content validity

In the 1st round, the content validity of 36 preliminary items was assessed. Four items for physical vitality (“I have a hunched-over body posture,” “My eyes are sore and dim,” “I feel light-footed,” and “My breathing is deep and even”) and three items for psychological vitality (“I want to stay still without moving,” ““I enjoy meeting new people,” and “I usually pay attention to the sounds and smells around me”) were deleted because of low I-CVI and redundancy. Three items for physical vitality and one item for psychological vitality were revised. In the 1st round, four preliminary items (two items for physical vitality and two items for psychological vitality) were added based on expert opinions.

In the 2nd round, the content validity of eight revised and added preliminary items (five items for physical vitality and three items for psychological vitality) was rated. One item for physical vitality (“I look forward to every meal”) was deleted because of low I-CVI.

As a result of the two rounds, a pool of 32 preliminary items, consisting of 16 physical and 16 psychological vitality items, was selected. The content validity of each item is presented in Table 1.

**Table 1:** Item analysis and I-CVI of the Integrative Vitality Scale.

Preliminary item	Min.	Max.	M	SD	Skew	Kurtosis	Item-total correlation	Internal consistency when items are deleted	I-CVI
No.1	0	4	1.88	0.86	0.10	−0.05	0.71	0.948	1.00
No.2	0	4	2.06	0.87	0.15	−0.23	0.72	0.948	0.90
No.3	0	4	2.30	0.90	−0.18	−0.16	0.58	0.949	1.00
No.4	0	4	1.76	1.00	0.03	−0.55	0.65	0.949	1.00
No.5	0	4	1.73	1.02	0.24	−0.66	0.53	0.950	0.90
No.6	0	4	1.89	0.87	0.00	−0.20	0.74	0.948	1.00
No.7^a^	0	4	2.36	0.99	−0.41	−0.50	0.52	0.950	0.70
No.8	0	4	2.58	0.82	−0.45	0.27	0.61	0.949	1.00
No.9	0	4	2.21	0.97	−0.34	−0.31	0.42	0.951	1.00
No.10	0	4	2.07	0.88	−0.12	−0.45	0.52	0.950	1.00
No.11	0	4	2.20	0.89	−0.02	−0.41	0.63	0.949	0.90
No.12	0	4	2.41	1.00	−0.34	−0.58	0.59	0.949	1.00
No.13	0	4	1.97	1.06	−0.05	−0.87	0.59	0.949	1.00
No.14	0	4	2.01	1.01	0.01	−0.59	0.61	0.949	0.90
No.15	0	4	2.10	0.91	−0.03	−0.21	0.67	0.949	1.00
No.16^a^	0	4	2.75	0.86	−0.42	−0.26	0.49	0.950	0.70
No.17	0	4	2.22	0.80	−0.16	−0.25	0.68	0.949	0.80
No.18	0	4	2.34	0.81	−0.27	0.16	0.75	0.948	1.00
No.19	0	4	2.39	0.84	−0.18	−0.15	0.74	0.948	1.00
No.20	0	4	2.30	0.86	−0.29	−0.06	0.64	0.949	1.00
No.21	0	4	2.17	0.85	−0.14	−0.03	0.68	0.949	1.00
No.22	0	4	2.54	0.89	−0.42	−0.30	0.46	0.950	0.90
No.23	0	4	2.54	0.87	−0.25	−0.51	0.56	0.950	0.90
No.24	1	4	2.77	0.73	−0.33	0.03	0.45	0.950	1.00
No.25	0	4	2.74	0.76	−0.52	0.37	0.62	0.949	1.00
No.26	1	4	2.80	0.75	−0.47	0.18	0.53	0.950	1.00
No.27^a^	0	4	2.05	0.98	−0.09	−0.55	0.58	0.949	0.80
No.28	0	4	2.37	0.84	−0.23	−0.22	0.71	0.948	1.00
No.29	0	4	2.32	0.92	−0.21	−0.34	0.68	0.948	1.00
No.30	0	4	2.23	0.88	−0.20	−0.51	0.56	0.949	0.80
No.31	0	4	2.54	0.87	−0.48	0.04	0.63	0.949	0.90
No.32	0	4	2.55	0.81	−0.33	0.06	0.41	0.951	0.80

I-CVI, item-level content validity index. ^a^Reversed item.

After EFA, the S-CVI/Ave of the final version of the IVS was calculated. The S-CVI/Ave of the IVS-total was 0.93, IVS-physical was 0.94, and IVS-psychological was 0.93. This suggests that both the IVS and its subscales have excellent content validity (38).

### 3.2 Item analysis

The results of the item analysis are presented in Table 1. The average (*SD*) of each item was 1.73 to 2.80 (0.73 to 1.06), which was acceptable. Each item had a skewness of <2 and kurtosis of <7, which were acceptable levels for achieving normality. The response range for two items (Nos. 24 and 26) ranged from 1 to 4, with no response to 0. Three items (Nos. 6, 13, and 18) had high inter-item correlations with other items, suggesting that the items were redundant. One item (No. 9) had a low inter-item correlation with the other items, suggesting that the item did not adequately represent the concept. The item-total correlation of all items was good, above 0.30 and below 0.80. When deleted, none of the items improved the overall internal consistency of the scale. In addition, one item (No. 22) did not meet the operational definition of psychological vitality.

In summary, 7 of the 32 preliminary items were deleted. Subsequently, 25 preliminary items were included in the EFA.

### 3.3 Exploratory factor analysis

Sample 1 for EFA was found to be adequate according to two criteria: the Kaiser-Meyer-Olkin (KMO) measure of sampling adequacy was higher than 0.80 (64, 65) and Bartlett’s Test of Sphericity was *p* < 0.05 (66). In Sample 1, the KMO measure of sampling adequacy was 0.95 and Bartlett’s test was *χ^2^*(300) = 4561.26, *p* < 0.001.

Eigenvalues, scree plot, and interpretability were used to determine the number of factors. Three, two, and two factors were found to be appropriate in the eigenvalues, scree plot, and interpretability. After setting up two factors, the loadings of all items were found to be good, above 0.30. However, three items (Nos. 3, 16, and 27) were deleted because they were cross-loaded on Factors 1 and 2 with a loading difference of less than 0.10.

As a result of the EFA, an IVS consisting of 22 items was developed. The IVS consisted of two subfactors: physical vitality and psychological vitality. Physical vitality consisted of 11 items, including 1 reversed item. Psychological vitality consisted of 11 items and did not include reversed items. The EFA results are listed in Table 2.

**Table 2:** Factor structure matrix with communalities of each item and eigenvalues, and % of variance accounted for each factor.

Preliminary item	Content	Factor		Communality coefficient
		1	2	
No.2	I feel refreshed.	**0.77**	−0.57	0.60
No.1	My body is full of energy.	**0.76**	−0.53	0.59
No.15	My limbs feel strong and light.	**0.76**	−0.49	0.59
No.4	I feel rejuvenated when I wake up in the morning.	**0.74**	−0.43	0.53
No.11	My chest feels clear and fresh.	**0.70**	−0.45	0.49
No.8	My breathing feels comfortable.	**0.68**	−0.47	0.48
No.14	I am full of energy even if I go out for two days in a row (excluding going out for work or study).	**0.67**	−0.45	0.49
No.12	I do not get tired even if I walk or stand for more than 30 min.	**0.66**	−0.44	0.48
No.5	My body does not feel stiff anywhere.	**0.65**	−0.33	0.46
No.10	My lower abdomen is warm.	**0.59**	−0.35	0.37
No.7^a^	My head feels heavy and achy.	**0.57**	−0.35	0.36
No.19	I have a passion for life.	0.58	**−0.81**	0.65
No.17	I am active and enthusiastic in everything I do.	0.50	**−0.76**	0.58
No.28	I am confident.	0.55	**−0.75**	0.59
No.21	I look forward to every new day.	0.52	**−0.74**	0.56
No.29	I feel hopeful about the future.	0.55	**−0.73**	0.58
No.20	I am usually cheerful.	0.48	**−0.73**	0.54
No.25	I am pleasantly immersed when I am doing something.	0.43	**−0.72**	0.54
No.23	I am excited by the idea of what I want to do.	0.34	**−0.70**	0.52
No.31	I find a positive side even in difficult situations.	0.48	**−0.69**	0.48
No.30	I find it interesting and fun even if something is repeated.	0.46	**−0.58**	0.40
No.32	I wonder about the meaning of the things I experience.	0.25	**−0.52**	0.31
Eigenvalues		9.60	2.22	
% of variance		43.65	10.11	

Factor 1 = physical vitality, factor 2 = psychological vitality. ^a^Reversed item. Bold text indicates which factor the item is included in.

### 3.4 Confirmatory factor analysis

**Figure 2:**
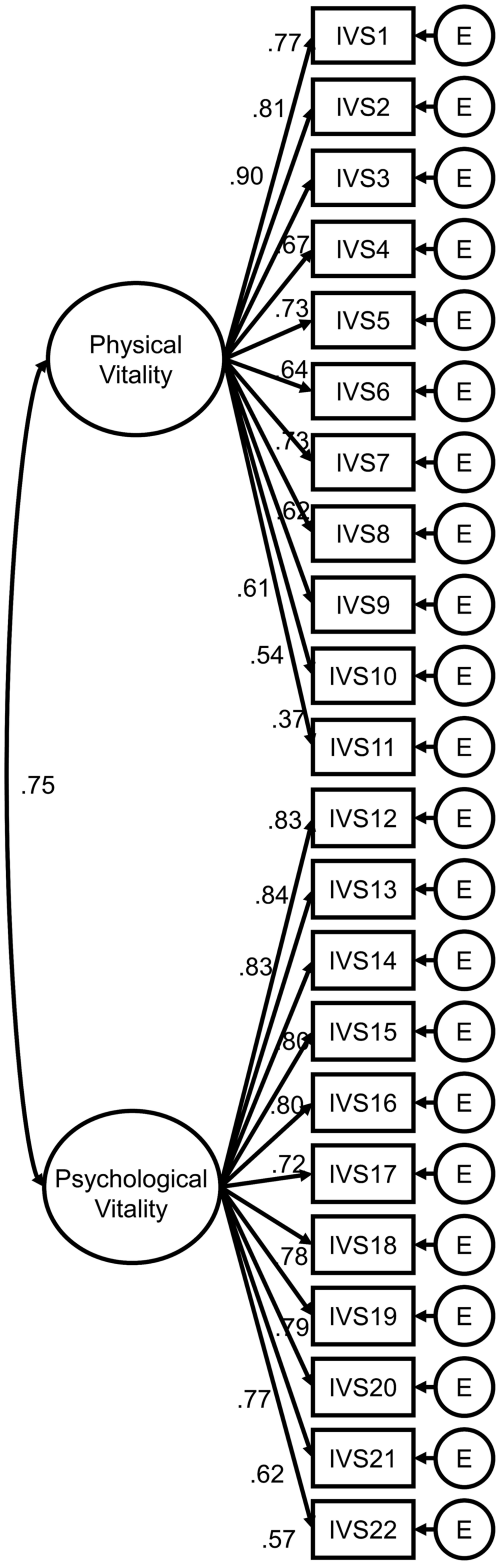
Confirmatory factor analysis of the two-factor model. The final 22-item Integrative Vitality Scale was analyzed. The standardized coefficients are presented. E = measurement error. All coefficients are significant at *p* < 0.001.

A CFA was conducted to confirm whether the two-factor structure of the IVS was appropriate. The two-factor model had an overall good model fit, *χ^2^*(208) = 552.81, *p* < 0.001; CFI = 0.926; TLI = 0.917; RMSEA = 0.069; SRMR = 0.045. The standardized coefficients of the observed variables ranged from 0.37 to 0.90. The correlation coefficient between physical and psychological vitalities was 0.75. The two-factor model of the IVS is presented in Figure 2.

### 3.5 Convergent and discriminant validity

To investigate the convergent validity of the IVS, a correlation analysis with the SVS and SF-36-VIT was conducted. Correlation analysis was conducted with the BPNSS and K-HCL-32 to investigate the discriminant validity of the IVS. Table 3 presents the results of correlation analysis. The criteria for interpreting the correlation coefficient are as follows: a coefficient less than 0.30 is a very low correlation, coefficient between 0.30 and 0.50 is a low correlation, coefficient between 0.50 and 0.70 is a moderate correlation, coefficient between 0.70 and 0.90 is a high correlation, and coefficient higher than 0.90 is a very high correlation (67, 68).

**Table 3:** Convergent and discriminant validity of the Integrative Vitality Scale.

Measure	IVS-Total		IVS-Physical		IVS-Psychological	
	Sample 1	Sample 2	Sample 1	Sample 2	Sample 1	Sample 2
SVS	0.72		0.56		0.75	
SF-36-VIT		0.69		0.65		0.62
K-HCL-30		0.39		0.32		0.39
BPNSS-Total		0.63		0.49		0.66
BPNSS-Autonomy		0.36		0.29		0.37
BPNSS-Competence		0.66		0.50		0.70
BPNSS-Relatedness		0.52		0.41		0.55

IVS, Integrative Vitality Scale; SVS, Subjective Vitality Scale; SF-36-VIT, Short Form 36 Health Survey – Vitality subdomain; K-HCL-30, Korean version of the Hypomania Checklist-32; BPNSS, Korean version of the Basic Psychological Need Satisfaction Scale. All coefficients are significant at *p* < 0.001.

The IVS-total had a high positive correlation with the SVS. IVS-physical was moderately positively correlated with the SVS, whereas IVS-psychological was highly positively correlated with the SVS. The IVS-total, IVS-physical, and IVS-psychological were moderately positively correlated with the SF-36-VIT and had a low positive correlation with the K-HCL-32. This suggests that the IVS is an indicator of sustainable well-being that is different from hypomania. The IVS-total was moderately positively correlated with BPNSS-total, BPNSS-competence, and BPNSS-relatedness, whereas it had a low positive correlation with BPNSS-autonomy. The IVS-physical had a relatively low correlation with the BPNSS and its subscales compared with the IVS-psychological. The IVS-psychological was moderately positively correlated with BPNSS-total, BPNSS-competence, and BPNSS-relatedness, whereas the IVS-physical had a low positive correlation with BPNSS-total, BPNSS-competence, and BPNSS-relatedness. The IVS-psychological has a low positive correlation with the BPNSS-autonomy, whereas the IVS-physical has a very low positive correlation with the BPNSS-autonomy. This suggests that psychological vitality is more closely related to BPNSS than physical vitality.

### 3.6 Criterion validity

To investigate the criterion validity of the IVS, correlation analysis was conducted with the K-BDI, FS, COMOSWB, and WHOQOL. Table 4 presents the results of correlation analysis.

**Table 4:** Criterion validity of the Integrative Vitality Scale.

Measure	IVS-Total		IVS-Physical		IVS-Psychological	
	Sample 1	Sample 2	Sample 1	Sample 2	Sample 1	Sample 2
K-BDI	−0.62	−0.58	−0.60	−0.50	−0.52	−0.57
FS	−0.56	−0.57	−0.63	−0.56	−0.37	−0.50
COMOSWB-Total	0.76	0.75	0.66	0.65	0.71	0.72
COMOSWB-LS	0.65	0.68	0.54	0.56	0.63	0.68
COMOSWB-PE	0.71	0.74	0.62	0.65	0.66	0.70
COMOSWB-NE	−0.65	−0.45	−0.59	−0.41	−0.58	−0.42
WHOQOL-Total	0.79		0.72		0.71	
WHOQOL-Overall	0.69		0.68		0.57	
WHOQOL-Physical	0.74		0.74		0.60	
WHOQOL-Psychological	0.75		0.63		0.72	
WHOQOL-Social	0.55		0.46		0.54	
WHOQOL-Environment	0.64		0.56		0.60	

IVS, Integrative Vitality Scale; K-BDI, Korean version of the Beck Depression Inventory; FS, Fatigue Scale; COMOSWB, Concise Measure of Subjective Well-Being; LS, life satisfaction; PE, positive emotion; NE, negative emotion; WHOQOL, Korean version of the World Health Organization Quality of Life Scale Abbreviated version. All coefficients are significant at *p* < 0.001.

The IVS-total, IVS-physical, and IVS-psychological had moderate negative correlations with the K-BDI. The IVS-total and IVS-physical had moderate negative correlations with the FS, whereas the IVS-psychological had a low negative correlation with the FS. The IVS-total had a high positive correlation with the COMOSWB-total and positive emotions. The IVS-total was moderately positively correlated with life satisfaction. The IVS-total had a low to moderate negative correlation with negative emotions. The IVS-physical had a moderate positive correlation with COMOSWB-total, life satisfaction, and positive emotions, and a moderate negative correlation with negative emotions. The IVS-psychological had a high positive correlation with the COMOSWB-total and a moderate positive correlation with life satisfaction and positive emotions. IVS-psychological had a low to moderate negative correlation with negative emotions. The IVS-total, IVS-physical, and IVS-psychological had a high positive correlation with the WHOQOL-total. The IVS-physical had a high positive correlation with WHOQOL-physical health, and IVS-psychological had a high positive correlation with WHOQOL-psychological health.

### 3.7 Incremental validity

Hierarchical regression analysis was conducted to investigate the incremental validity of the IVS relative to the SVS. The SVS was added as a predictor in Step 1, and the IVS was added as a predictor in Step 2. Table 5 presents the regression analysis results. In the case of depression, when IVS was added to Step 2, the predictive power for depression was significantly increased compared with that in Step 1. In Step 2, the SVS did not predict depression, whereas the IVS significantly predicted depression. In the case of fatigue, when IVS was added to Step 2, the predictive power for fatigue was significantly increased compared with that in Step 1. In Step 2, the SVS did not predict fatigue, whereas the IVS significantly predicted fatigue. In the case of subjective well-being, when IVS was added to Step 2, the predictive power for subjective well-being significantly increased compared with that in Step 1. In Step 2, both IVS and SVS significantly predicted subjective well-being.

**Table 5:** Incremental validity of the Integrative Vitality Scale (compared with the SVS).

IV	Step	DV	*B*	*SE*	*β*	*t*	*R* ^2^	*ΔR* ^2^	*F*
K-BDI	Step 1	SVS	−0.98	0.09	−0.50	−10.60^***^	0.25	0.25	112.39^***^
	Step 2	SVS	−0.19	0.12	−0.10	−1.58	0.39	0.14	110.66^***^
		IVS	−0.38	0.04	−0.55	−9.08^***^			
FS	Step 1	SVS	−0.69	0.08	−0.41	−8.42^***^	0.17	0.17	70.92^***^
	Step 2	SVS	−0.03	0.11	−0.02	−0.29	0.31	0.14	78.28^***^
		IVS	−0.32	0.04	−0.55	−8.44^***^			
COMOSWB	Step 1	SVS	1.34	0.09	0.63	14.96^***^	0.39	0.39	223.80^***^
	Step 2	SVS	0.35	0.11	0.17	3.31^**^	0.59	0.20	245.50^***^
		IVS	0.47	0.04	0.64	12.75^***^			

IV, independent variable; DV, dependent variable; IVS, Integrative Vitality Scale; SVS, Subjective Vitality Scale; K-BDI, Korean version of the Beck Depression Inventory; FS, Fatigue Scale; COMOSWB, Concise Measure of Subjective Well-Being. ^**^p < 0.01, ^***^p < 0.001.

Hierarchical regression analysis was conducted to investigate the incremental validity of the IVS relative to the SF-36-VIT. In Step 1, the SF-36-VIT was added as a predictive variable, and in Step 2, the IVS was added as a predictive variable. Table 6 presents the regression analysis results. In the case of depression, when IVS was added to Step 2, the predictive power for depression was significantly increased compared with that in Step 1. In Step 2, both the IVS and SF-36-VIT significantly predicted depression. In the case of fatigue, when IVS was added to Step 2, the predictive power for fatigue was significantly increased compared with that in Step 1. In Step 2, both the IVS and SF-36-VIT significantly predicted fatigue. In the case of subjective well-being, when IVS was added to Step 2, the predictive power for subjective well-being significantly increased compared with that in Step 1. In Step 2, both IVS and SF-36-VIT significantly predicted subjective well-being.

**Table 6:** Incremental validity of the Integrative Vitality Scale (compared with the SF-36-VIT).

IV	Step	DV	*B*	*SE*	*β*	*t*	*R* ^2^	*ΔR* ^2^	*F*
K-BDI	Step 1	SF-36-VIT	−1.38	0.11	−0.55	−12.25^***^	0.30	0.30	150.13^***^
	Step 2	SF-36-VIT	−0.79	0.15	−0.28	−4.74^***^	0.38	0.08	106.51^***^
		IVS	−0.26	0.04	−0.39	−6.65^***^			
FS	Step 1	SF-36-VIT	−1.36	0.09	−0.62	−14.80^***^	0.39	0.39	219.06^***^
	Step 2	SF-36-VIT	−0.95	0.12	−0.43	−7.67^***^	0.43	0.04	127.89^***^
		IVS	−0.16	0.03	−0.27	−4.79^***^			
COMOSWB	Step 1	SF-36-VIT	1.78	0.11	0.67	16.69^***^	0.44	0.44	278.55^***^
	Step 2	SF-36-VIT	0.76	0.13	0.29	6.06^***^	0.60	0.16	262.21^***^
		IVS	0.39	0.03	0.55	11.70^***^			

IV, independent variable; DV, dependent variable; IVS, Integrative Vitality Scale; SF-36-VIT, Short Form 36 Health Survey – Vitality subdomain; K-BDI, Korean version of the Beck Depression Inventory; FS, Fatigue Scale; COMOSWB, Concise Measure of Subjective Well-Being. ***p < 0.001.

### 3.8 Structural equation model

The goodness of fit of the research and competition models is presented in Table 7. The fit of the research model was excellent in terms of CFI, TLI, RMSEA, and SRMR (69). In contrast, the competition model showed excellent CFI and TLI, good SRMR, and poor RMSEA (69). As a result of the *χ^2^* difference test, *Δχ^2^* = 55.05 (*Δdf* = 1, *p* = 0.000), the research model was found to be more suitable than the competition model.

**Table 7:** Goodness of fit of structural equation model (research model vs. competition model).

	*χ* ^2^	*df*	CFI	TLI	RMSEA	SRMR
Research model	38.20^*^	24	0.994	0.991	0.041	0.022
Competition model	93.25^***^	25	0.971	0.959	0.089	0.054

^*^p < 0.05, ^***^p < 0.001.

**Figure 3:**
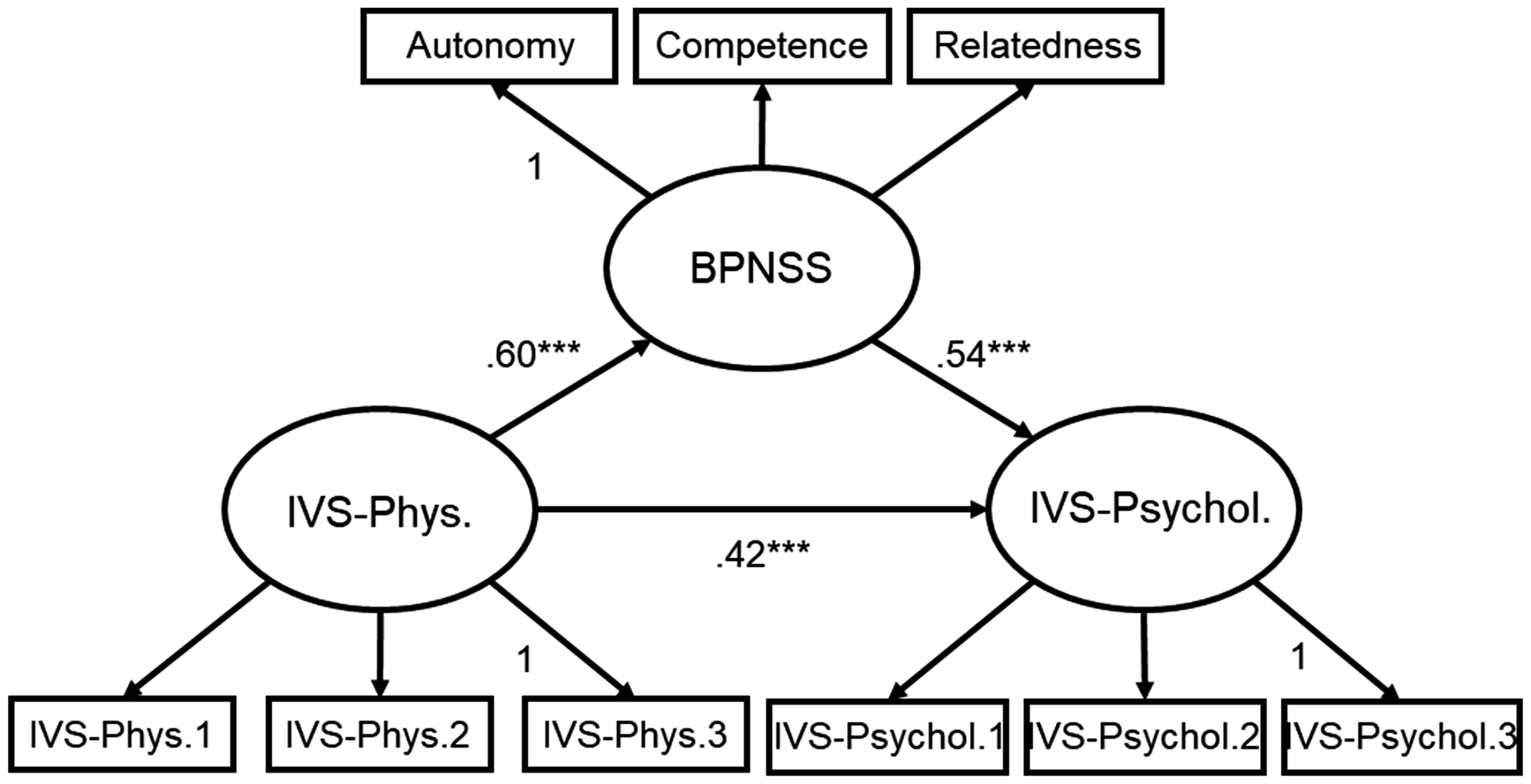
Standardized path coefficient of the research model. IVS-Phys., Integrative Vitality Scale-Physical vitality; IVS-Psychol., Integrative Vitality Scale-Psychological vitality; BPNSS, Korean version of the Basic Psychological Need Satisfaction Scale. ^***^p < 0.001.

The standardized coefficients of the research model are presented in Figure 3, and all coefficients of the direct path were significant. The indirect path between physical vitality and psychological vitality was also significant, *β* = 0.32, *p* = 0.001, 95% CI = 0.30–0.55.

### 3.9 Internal consistency and test–retest reliability

In Sample 1, the Cronbach’s *α* for IVS-total was 0.94, for IVS-physical was 0.92, and for IVS-psychological was 0.91. In Sample 2, the Cronbach’s *α* for IVS-total was 0.94, 0.89 for IVS-physical, and 0.94 for IVS-psychological.

In Sample 2, two surveys were conducted at two-week intervals. The correlation coefficient across timepoints was 0.78 for IVS-total, 0.71 for IVS-physical, and 0.80 for IVS-psychological.

### 3.10 Pre-post comparison

The results of the 16-week mindfulness training are presented in Table 8. Compared to the pre-test, mindfulness training significantly improved IVS, PWBS, and CAMS-R, but not COMOSWB, IVS: *p* = 0.007; PWBS: *p* = 0.012; CAMS-R: *p* = 0.026; COMOSWB: *p* = 0.871.

**Table 8:** Comparison of mean (SD) before and after mindfulness training.

	Pre (*n* = 28)	Post (*n* = 28)	*t*
IVS-Total	49.11 (12.14)	55.92 (12.71)	−2.936^**^
IVS-Physical	22.39 (6.87)	26.54 (6.78)	−2.843^**^
IVS-Psychological	26.71 (7.60)	29.38 (7.22)	−2.502^*^
COMOSWB	19.03 (8.21)	19.22 (9.62)	−0.164
COMOSWB-LS	15.12 (2.95)	15.30 (3.64)	−0.360
COMOSWB-PE	14.09 (3.21)	14.30 (3.43)	−0.359
COMOSWB-NE	10.18 (4.23)	10.37 (3.86)	−0.260
PWBS	184.32 (28.08)	192.57 (29.33)	−2.684^*^
PWBS-SA	59.93 (9.78)	61.11 (9.72)	−1.014
PWBS-AG	33.71 (10.53)	37.07 (9.82)	−2.488^*^
PWBS-AT	31.57 (7.59)	32.75 (7.39)	−1.531
PWBS-AL	26.04 (4.89)	27.32 (4.46)	−1.983
PWBS-ST	19.39 (2.04)	19.18 (2.11)	−0.605
PWBS-AD	13.68 (4.64)	15.14 (3.24)	−2.096^*^
CAMS-R	24.96 (5.32)	26.79 (5.31)	−2.355^*^

IVS, Integrative Vitality Scale; COMOSWB, Concise Measure of Subjective Well-Being; LS, life satisfaction; PE, positive emotion; NE, negative emotion; PWBS, Psychological Well-Being Scale; SA, self-acceptance; AG, awareness of goal; AT, autonomy; AL, activeness of life; ST, self-transcendence; AD, adventure; CAMS-R, Cognitive and Affective Mindfulness Scale-Revised. ^*^p < 0.05, ^**^p < 0.01.

## 4 Discussion

This study developed and validated a tool to evaluate integrative vitality based on TEM and modern psychology. Finally, the IVS was developed, which consisted of 22 items in two subfactors. These two subfactors were named physical and psychological vitality. Physical vitality is operationally defined as a positive physical experience in a state of relaxation and rest and consists of 11 items, including one reversed item. Psychological vitality is operationally defined as a state of being intrinsically motivated, feeling interest and fun in life, and being active; it consists of 11 items without reversed items. The IVS was found to have good internal consistency and test–retest reliability.

As a result of the content validity evaluation, most items in the final version of the IVS had an I-CVI ≥ 0.80, and the overall IVS and subscales had an S-CVI/Ave ≥ 0.90. When I-CVI ≥ 0.78, it corresponds to good content validity (37), and when S-CVI/Ave ≥ 0.90, it corresponds to excellent content validity (38). Accordingly, the IVS has good content validity, suggesting that it is suitable for evaluating health-related variables such as vitality.

As a result of the CFA, the two-factor model of the IVS had a good fit, CFI = 0.926, TLI = 0.917, RMSEA = 0.069, SRMR = 0.045. According to Vandenberg and Lance (70), CFI and TLI are considered good fits when they are above 0.90, and RMSEA and SRMR are considered good fits when they are below 0.08. These results suggest that the two-factor IVS model is validated with empirical support.

Correlation analysis showed that the IVS had a high positive correlation with the SVS and a moderate positive correlation with the SF-36-VIT. The SVS is a scale developed and validated to evaluate subjective vitality, and the SF-36-VIT is a validated scale that includes various subdomains, such as vitality, to measure quality of life. These results suggest that the IVS evaluates the vitality construct similarly to existing scales and support the convergent validity of the IVS.

The IVS had a low positive correlation with the K-HCL-32. Mania or hypomania is a symptom that alternates with depression in bipolar disorder and refers to persistent and excessive levels of energy. Mania includes exaggerated self-confidence, reduced need for sleep, jumps in thought processes, distractions, and excessive preoccupation with pleasurable activities (71). In contrast, integrative vitality is distinct from mania in that it emphasizes sufficient relaxation and rest. Additionally, because integrative vitality is an intrinsically motivated state based on autonomy, it can be distinguished from an impulsive state that is excessively obsessed with external rewards. This suggests that integrative vitality is an indicator of sustainable well-being, which is different from hypomania.

Physical vitality had a relatively low positive correlation with the overall BPNSS and its subscales compared with psychological vitality. These results are consistent with the SDT, which states that intrinsic motivation is induced when basic psychological needs such as autonomy, competence, and relatedness are satisfied (31, 32). According to the SDT, psychological vitality, operationally defined as an intrinsically motivated state, is highly correlated with the satisfaction of basic psychological needs. Moreover, the results of this study are consistent with the hierarchy of needs theory (57). Physical vitality is a state in which physiological resources are restored through relaxation and rest and may be a prerequisite for pursuing higher-level basic psychological needs. However, satisfying physiological needs does not directly lead to satisfaction of basic psychological needs. Therefore, physical vitality is expected to have a lower correlation with satisfaction of basic psychological needs compared with psychological vitality. Psychological vitality had a moderately positive correlation with the overall BPNSS and subscales, whereas physical vitality had a low to very low positive correlation with the overall BPNSS and subscales. These results suggest that physical and psychological vitalities are distinct concepts.

The IVS was moderately negatively correlated with the K-BDI and FS. Depression is a mental health problem characterized by a depressed mood and the absence of positive emotions such as interest and pleasure (71). It is a mental illness that negatively affects not only an individual’s quality of life but also overall occupational and social functioning (72). Fatigue is the depletion of physiological and psychological resources due to excessive energy utilization (73, 74). At this time, various unpleasant symptoms such as tiredness and decreased motivation are experienced (75, 76). Depression and fatigue have common symptoms of decreased physical and psychological energy. Physical vitality refers to a healthy energy balance in which physical resources are restored through relaxation and rest, and psychological vitality refers to intrinsic motivation. Accordingly, integrative vitality provides sufficient physical and psychological energy, which is the opposite of depression and fatigue. These results suggest that the IVS can predict energy-related symptoms and disorders, such as depression and fatigue.

The IVS was highly positively correlated with the WHOQOL and COMOSWB. These results are consistent with those of previous studies suggesting that vitality is a health-related concept closely related to quality of life and well-being (21). In addition, physical vitality was highly positively correlated with WHOQOL-physical health, whereas psychological vitality was highly positively correlated with WHOQOL-psychological health, which is consistent with previous studies suggesting that vitality is included in the health-related area among various areas of quality of life (21). The IVS was highly correlated with COMOSWB-positive emotions, whereas it had a low to moderate correlation with COMOSWB-negative emotions. This finding suggests that integrative vitality is a positive feeling associated with relaxation, contentment, interest, and joy. These results suggest that the IVS can predict quality of life and well-being.

Hierarchical regression analysis showed that the IVS significantly predicted the K-BDI, FS, and COMOSWB, even after controlling for the SVS and SF-36-VIT. Both the SVS and SF-36-VIT have been developed and validated to measure subjective vitality, similar to the IVS. These results suggest that the IVS could provide additional predictive power for mental health issues such as depression, fatigue, and subjective well-being. Moreover, the regression coefficient of the IVS was significant for depression and fatigue, whereas that of the SVS was not. These results suggest that the IVS may replace the SVS in predicting depression and fatigue.

Structural equation modeling showed that the fit of the research model was better than that of the competition model. In other words, satisfaction with basic psychological needs partially mediated the relationship between physical and psychological vitality. According to the hierarchy of needs theory (57), physical vitality is the satisfaction of physiological needs through relaxation and rest and may be a preceding factor in the pursuit of basic psychological needs. According to the SDT (31, 32), intrinsic motivation is experienced when basic psychological needs are satisfied. Finally, according to broaden and build theory (77), physical vitality is a positive feeling that expands attention and induces active engagement in new environments. These results are consistent with those of psychological theories of need, motivation, and emotions.

The 16-week mindfulness training significantly improved IVS at post-training compared to pre. Mindfulness induces the relaxation response (23), which is associated with physical vitality. In addition, mindfulness enhances self-regulation and well-being through decentering (78, 79), which is associated with psychological vitality. In fact, previous studies have shown that mindfulness training improves vitality and well-being (80, 81). This finding suggests that IVS assesses physical and psychological well-being, and that IVS can be used as a measurement to investigate the effectiveness of mindfulness-based mind–body training such as meditation and yoga.

**Figure 4:**
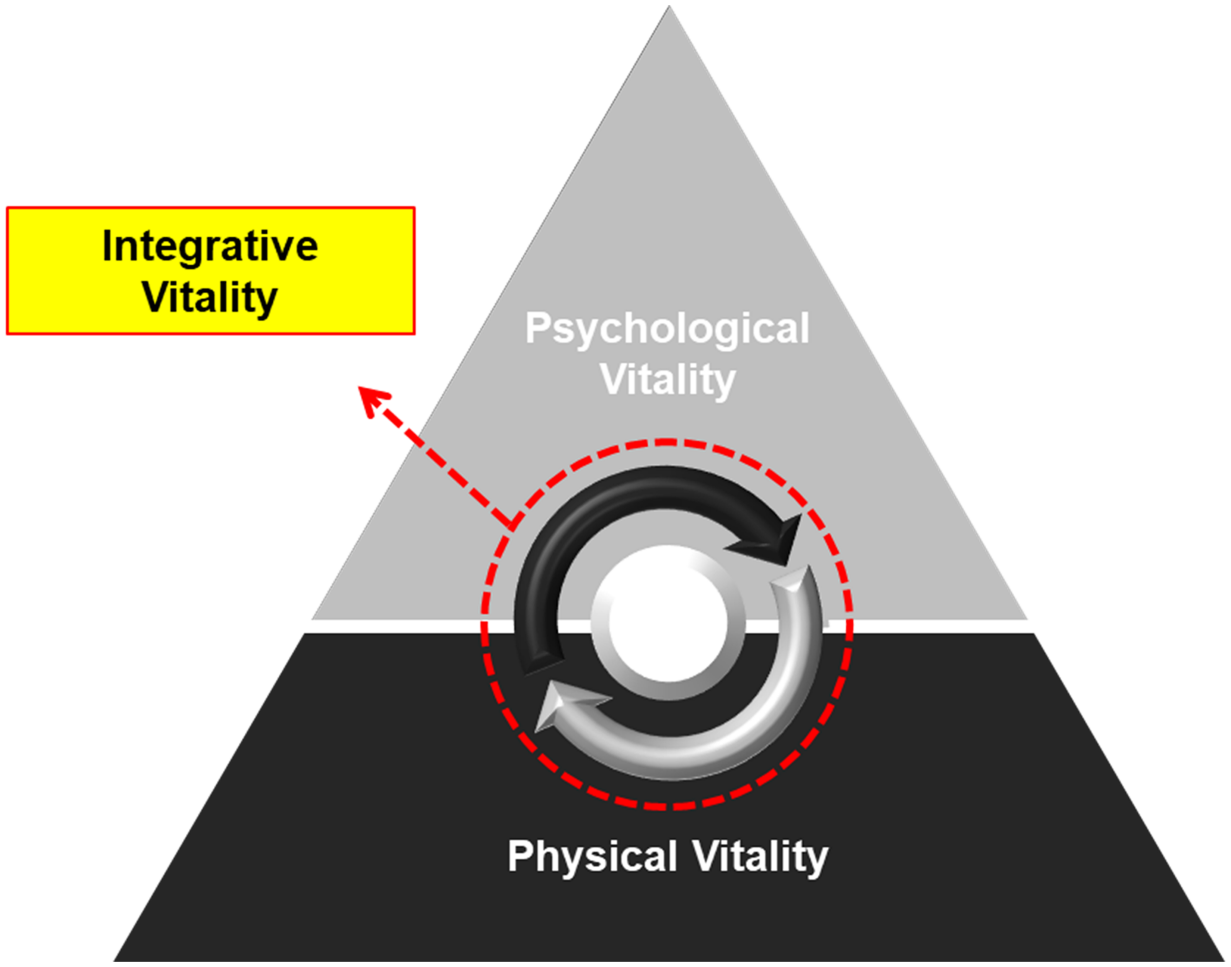
Conceptual model of integrative vitality. This figure illustrates the structure of integrative vitality. Physical vitality is a positive experience that is obtained through relaxation and rest. Psychological vitality is the intrinsically motivated state of being active, feeling interested, and having fun in life. This Pyramid model suggests that psychological vitality is based on physical vitality. The arrows within the dotted circle indicate that integrative vitality is established during the process of circulating physical and psychological vitality. Integrative vitality refers to a state in which both physical and psychological vitality are high, suggesting a balance and harmony between rest (relaxation) and activity (awakening).

An integrated view of vitality is shown in Figure 4. The pyramid model in Figure 4 does not imply a value hierarchy for physical and psychological vitality. Similar to the hierarchy of needs theory, psychological vitality appears only after physical vitality is satisfied. More importantly, this model implies that integrative vitality is established through the circulation of physical and psychological vitality. Integrative vitality is a state of sufficient physical and psychological vitality, that is, a balance between rest (relaxation) and activity (awakening). Integrative vitality cannot be solely established through physical or psychological vitality. Continuing activity without sufficient rest leads to exhaustion (e.g., general adaptation syndrome), and rest without activity is no different from losing the meaning of life (e.g., depression). This idea was inspired by the perspective of TEM, where balance and harmony are the core principles of healthcare, and circulation is the mechanism that induces balance and harmony (82).

### 4.1 Limitations

Although this study demonstrated the reliability and validity of the IVS, it had several limitations. First, further validation is required for the IVS to be used as an indicator of healthy aging. Vitality is an intrinsic capacity of all generations. To be used across all ages, the IVS was developed and validated with a variety of age samples from 20s to over 60s. However, due to the relatively small number of older adult samples, further studies targeting the older adult population are needed for the IVS to be utilized as an indicator of healthy aging.

Second, this study was not conducted in a clinical setting and did not involve any patients. This study found that the IVS is associated with symptoms and disorders related to low energy, such as depression and fatigue, and is highly related to an individual’s well-being and quality of life. Replicating the correlation between the IVS and clinical outcome measures in clinical settings provides a clear basis for the validity of the IVS in clinical settings. Therefore, further research is needed to investigate the relationship between the IVS and energy-related symptoms, such as frailty, depression, and chronic fatigue, in clinical settings.

Third, there may be a lack of items that address the degree of motivation in the psychological vitality sub-factor. According to self-determination theory, motivation is divided into extrinsic motivation and intrinsic motivation based on the level of autonomy, which refers to the locus of motivation. For example, responding with a high score to a certain item (e.g., ‘I am excited by the idea of what I want to do.’) means having intrinsic motivation rather than extrinsic motivation, but it may not mean how high the degree of motivation is.

Fourth, various factors affecting integrative vitality should be considered. A comparison of the IVS based on sex and age (Supplementary materials 2, 3) showed that men had significantly higher integrative and physical vitality than women. In terms of age, people in their 40s had significantly lower integrative, physical, and psychological vitality than people in their 60s. Interestingly, vitality continued to decrease from the 20s to the 40s but tended to increase again from the 50s. Factors that cause this nonlinearity may include socioeconomic status, presence of disease, and stressful events. Therefore, future research should explore the various factors that may affect integrative vitality.

Fifth, this study was conducted among adults in a Far East Asian country; therefore, cross-cultural generalization may not be possible. For example, physical vitality partly incorporates concepts from TEM (e.g., Qi), and it is unknown whether it would respond similarly in Western samples. Therefore, follow-up validation studies in Western countries are warranted.

Sixth, the generalizability of this study is limited because the IVS was developed and validated through an online survey. In addition, incentives provided in online surveys may cause sample bias. Therefore, future research should conduct a repeat validation of the IVS in offline fields.

### 4.2 Conclusion

The IVS was developed to assess physical and psychological vitality. The IVS had good internal consistency and test–retest reliability. The IVS assessed subjective vitality similar to the SVS and SF-36-VIT but had additional predictive power for depression, fatigue, and well-being compared with the SVS and SF-36-VIT. The IVS was an indicator of sustainable well-being that is differentiated from hypomania. The IVS can help predict not only symptoms related to low energy, such as depression and fatigue, but also overall health, such as well-being and quality of life.

## Data Availability

The raw data supporting the conclusions of this article will be made available by the authors, without undue reservation.
