# Supplementary material for: Development and validation of the Integrative Vitality Scale
Source: Front Public Health. 2024 Nov 18;12:1452068. doi: 10.3389/fpubh.2024.1452068 (PMC11609079; doi:10.3389/fpubh.2024.1452068)
Supplement: Supplementary file 1 [file Supplementary_file_1.docx]

Supplementary Material 1. The final version of the Integrative Vitality Scale

| The items below are things you could experience physically or psychologically in your daily life. Please read each item carefully and check how much it matches your usual experience over the past two weeks. (Likert scale 0 (not at all)-4(completely)) | | |
| --- | --- | --- |
| **#** | **item (Korean)** | **item (English)** |
| 1 | 상쾌한 느낌이 든다. | I feel refreshed. |
| 2 | 몸에 힘이 넘친다. | My body is full of energy. |
| 3 | 팔다리에 힘이 있고 가볍다. | My limbs feel strong and light. |
| 4 | 잠에서 깨어날 때 개운하다. | I feel rejuvenated when I wake up in the morning. |
| 5 | 가슴이 막힘없이 시원하다. | My chest feels clear and fresh. |
| 6 | 호흡이 편안하다. | My breathing feels comfortable. |
| 7 | 이틀 연속 외출해도 힘이 넘친다. (학업, 직업에 의한 외출 제외) | I am full of energy even if I go out for two days in a row (excluding going out for work or study). |
| 8 | 30분 이상 걷거나 서 있어도 지치지 않는다. | I don’t get tired even if I walk or stand for more than 30 minutes. |
| 9 | 몸에 결리는 부위가 없다. | My body doesn’t feel stiff anywhere. |
| 10 | 아랫배가 따뜻하다. | My lower abdomen is warm. |
| 11^a^ | 머리가 무겁고 아프다. | My head feels heavy and achy. |
| 12 | 인생을 살아가는 열정이 있다. | I have a passion for life. |
| 13 | 매사에 적극적이다. | I am active and enthusiastic in everything I do. |
| 14 | 자신감이 있다. | I am confident. |
| 15 | 매번 새로운 날이 기대된다. | I look forward to every new day. |
| 16 | 미래에 대해 희망적으로 느낀다. | I feel hopeful about the future. |
| 17 | 평소에 쾌활하다. | I am usually cheerful. |
| 18 | 무언가를 하고 있을 때 즐겁게 몰입한다. | I am pleasantly immersed when I am doing something. |
| 19 | 하고 싶은 것에 대한 생각으로 흥미진진하다. | I am excited by the idea of what I want to do. |
| 20 | 어려운 상황에서도 긍정적인 측면을 발견한다. | I find a positive side even in difficult situations. |
| 21 | 반복되는 일이라도 흥미와 재미를 느낀다. | I find it interesting and fun even if something is repeated. |
| 22 | 내가 경험하는 것들의 의미가 궁금하다. | I wonder about the meaning of the things I experience. |
| The development and validation of this scale were conducted with Korean version. The English version were translated from Korean into English by a psychologist who is bilingual in English and Korean.  ^a^ reversed item | | |
