# Supplementary material for: Development and validation of the Integrative Vitality Scale
Source: Front Public Health. 2024 Nov 18;12:1452068. doi: 10.3389/fpubh.2024.1452068 (PMC11609079; doi:10.3389/fpubh.2024.1452068)
Supplement: Supplementary file 2 [file Supplementary_file_2.docx]

Supplementary Material 2. Mean differences in IVS by gender combining Sample 1 and 2

|  | **Male (*n* = 348)** | **Female (*n* = 349)** | ***t*** | ***p*** |
| --- | --- | --- | --- | --- |
| Integrative Vitality | 48.3 (13.6) | 46.1 (13.2) | 2.23 | .026 |
| Physical Vitality | 23.0 (7.4) | 21.3 (7.1) | 3.24 | .001 |
| Psychological Vitality | 25.3 (7.4) | 24.8 (7.6) | 0.87 | .383 |
| This is the result of a comprehensive analysis of Samples 1 and 2. | | | | |
