# Supplementary material for: Development and validation of the Integrative Vitality Scale
Source: Front Public Health. 2024 Nov 18;12:1452068. doi: 10.3389/fpubh.2024.1452068 (PMC11609079; doi:10.3389/fpubh.2024.1452068)
Supplement: Supplementary file 3 [file Supplementary_file_3.docx]

Supplementary Material 3. Mean differences in IVS by age combining Sample 1 and 2

|  | **20’s** | **30’s** | **40’s** | **50’s** | **Over 60’s** | ***F*** | ***p*** |
| --- | --- | --- | --- | --- | --- | --- | --- |
| *n* | 140 | 139 | 139 | 139 | 140 |  |  |
| Integrative Vitality | 47.67 (12.6) | 46.3 (14.9) | 43.8_a_ (13.0) | 47.6 (13.2) | 50.6_b_  (12.8) | 4.79 | < .001 |
| Physical Vitality | 22.5  (7.0) | 21.5  (8.0) | 20.3_a_  (7.1) | 22.3  (6.9) | 24.1_b_  (7.0) | 5.27 | < .001 |
| Psychological Vitality | 25.2  (7.4) | 24.8  (8.2) | 23.5_a_  (7.3) | 25.3  (7.3) | 26.5_b_  (6.8) | 2.93 | .020 |
| *Note.* This is the result of a comprehensive analysis of Samples 1 and 2.  Means with different subscripts are significantly differ at the *p* = .05 according to Scheffe test procedure. | | | | | | | |
